# Does mindfulness help employees express ethical voice? a moderated mediation model of mindfulness and ethical voice

**DOI:** 10.1186/s40359-026-04206-0

**Published:** 2026-02-26

**Authors:** Kaixin Zhang, Zilong Cui

**Affiliations:** 1 School of Management, Changchun Guanghua University, No.3555 Wuhan Road, Changchun, China; 2 School of Business Administration, Jilin University of Finance and Economics, No.3699, Jing Yue Street, Changchun, China

**Keywords:** Mindfulness, Ethical voice, Moral self-efficacy, Moral courage, Moderated mediation

## Abstract

Following a series of major ethical scandals in various organizations, researchers have increasingly sought to understand the role of ethical voice in the workplace. Existing ethical voice research centres on leadership-focused models and overlooks the individual psychological mechanisms connecting traits to ethical voice, creating a key gap in understanding the micro-foundations of such behaviour. The results of a longitudinal field study featuring 453 participants from one Chinese firm were used to investigate the model hypothesized in this research. The results reveal that employee mindfulness has a positive effect on ethical voice via moral self-efficacy and moral courage. Moreover, a caring ethical climate strengthens the relationship between mindfulness and ethical voice via moral self-efficacy and moral courage. The findings of this study contribute to the literature on both mindfulness and ethical voice. The findings of this research also have important practical implications for organizations, which can enable them to develop more appropriate human resource management strategies that can promote ethical voice in the workplace; these findings also offer guidance regarding how to manage this entire process effectively.

## Introduction

Unethical behaviour and ethical violations in the workplace entail a substantial threat to firms, particularly following several high-profile corporate crises; in turn, these behaviours convey negative images of organizations [1–3]. Given these ethical crises and the existence of higher ethical standards in the workplace, the need for ethical behaviour on the part of organization members has become increasingly vital at work [4, 5]. For example, organization members can target unethical matters within the organization by engaging in voice behaviours, which may provide the organization with a timely way to avoid potential ethical crises. This kind of voice, which focuses on moral issues in organizations, has been the subject of great concern in recent studies [6, 7]. Ethical voice is defined in terms of the communication by individual organization members of concerns regarding violations of societal ethical standards (e.g., honesty, fairness, care, respect) and/or suggestions concerning the need to uphold societal ethical standards [7]. As a subset of general voice—voluntary acts to improve organizational operations [8]—ethical voice is uniquely anchored in ethical norms, with moral integrity as its core motivation, distinct from general voice’s focus on broad efficiency; compared to promotive and prohibitive voice (Liang et al., 2012), it overlaps with the latter in opposing unethical acts but exclusively targets ethical issues (unlike prohibitive voice’s potential focus on non-ethical inefficiencies) and can be promotive yet guided by ethical, not performance, goals. In summary, its uniqueness lies in an ethical-centric orientation, upholding moral standards and anchoring our framework in the ethical dimension of voice. Ethical voice has received a great deal of attention recently due to its unique pro-social characteristics [9–11]. However, despite the increasing attention that scholars have devoted to the connection between contextual factors and ethical voice, for example, previous research on this topic has focused primarily on how leader-related factors motivate ethical voice [1, 11]; accordingly, the theoretical background of the ability of individual factors to motivate ethical voice has been overlooked in the literature to some degree. Specifically, individual factors are essential facilitators of an individual’s voice within an organization [12]. Thus, a significant research gap lies in the fact that research in this field has yet to produce a systematic examination of how and why individual factors may influence ethical voice.

Mindfulness refers to the individual’s receptive attention to and awareness of present events and experiences [13]. Mindfulness has generally been viewed as trait-like, such that individuals may exhibit high or low (or medium) levels of mindfulness that are stable across time [14]. DJ Good, CJ Lyddy, TM Glomb, JE Bono, KW Brown, MK Duffy, RA Baer, JA Brewer and SW Lazar [15] claimed that mindfulness is an experience-based mode of mind rather than a conceptual one. In this experiential mode, individuals focus on their current experiences rather than prior thoughts or notions. In recent years, individual mindfulness has been regarded as an important individual factor driving individuals to engage in moral behavior [16]. Specifically, mindfulness increases one’s awareness of one’s surroundings, including with regard to ethical concerns [17, 18], thus making one more likely to notice ethical difficulties or value conflicts of interest [19]. Despite this, mindfulness is believed to help individuals engage in voice behaviour in the workplace [20]. For example, mindful employees accept the risk of using their constructive voice objectively rather than interpreting the possible hazards subjectively as either right or wrong (or good or terrible) [21]. Accordingly, does the ethically focused character of mindfulness promote ethical voice in the workplace? What mechanisms underlie this effect? If trait mindfulness can motivate ethical voice in an organization, then training individuals in mindfulness can ultimately help increase the ethical standards of the organization. On this basis, this study intends to fill this research gap and extend previous studies by exploring the impact of mindfulness on ethical voice as well as the corresponding internal mechanisms and boundary conditions. To synthesize the core objectives of this study, we propose the following overarching research question: How do individual traits (mindfulness, moral self-efficacy, moral courage) interact with a contextual factor (caring ethical climate) to influence employees’ ethical voice behavior, and what are the mediating mechanisms underlying these relationships?

Ethical voice is essentially a moral behavior that individuals engage in when encountering a moral issue, and it results from individuals’ moral decision-making when faced with multiple possible options [22]. Hence, to investigate the mechanism underlying the link between mindfulness and ethical voice, we employ a moral decision-making model [23, 24]. According to moral decision-making model [23, 24], individual factors influence an individual’s moral cognition and moral emotions, thereby triggering subsequent moral acts. We suggest mindfulness can motivate employees to conceive solutions to ethical dilemmas, and increase their moral self-efficacy (individuals’ subjective beliefs and self-judgments regarding their capability to sensitively identify ethical conflicts, adhere to moral principles and organizational norms, resist unethical temptations, and successfully engage in ethically congruent behaviours) [25], thereby facilitating the implementation of their ethical voice [26]. Trait mindfulness has also been suggested to foster moral emotions while upholding moral principles and is believed to catalyse individual moral courage [27]. The moral courage that is developed alongside mindfulness also facilitates the individual’s implementation of ethical voice [28]. Therefore, exploring the mediating roles of moral self-efficacy and moral courage in the relationship between mindfulness and ethical voice is the second research aim of this study.

**Fig. 1:**
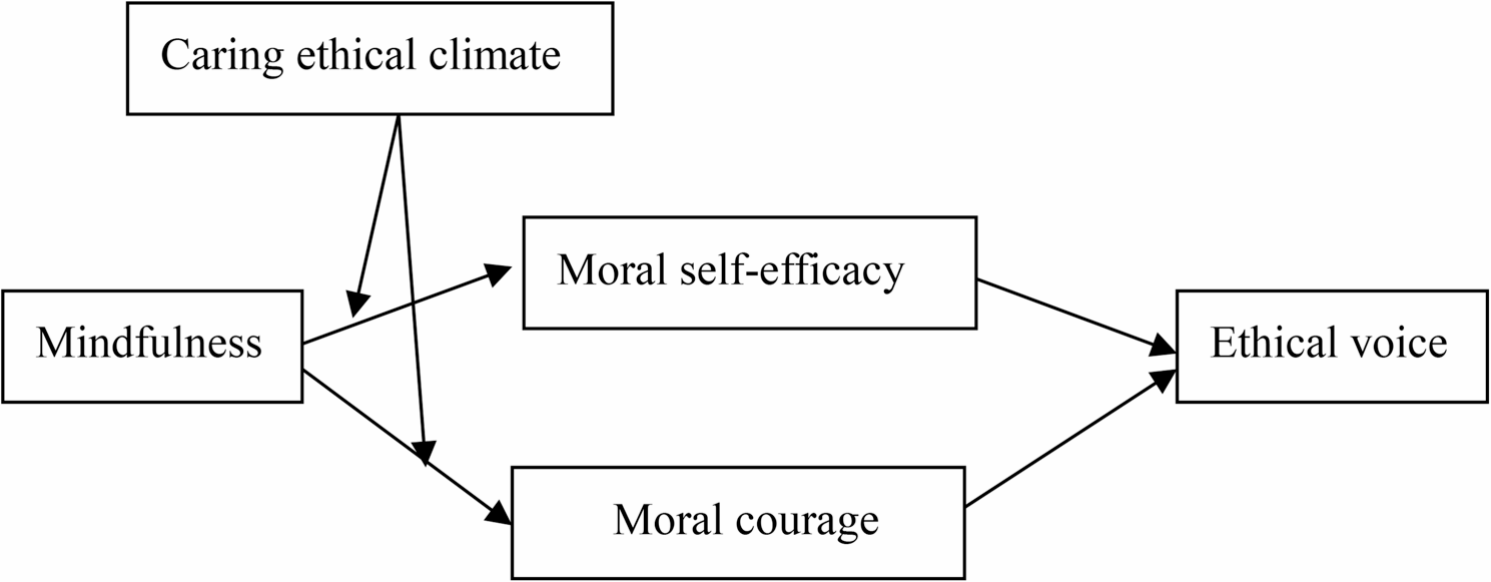
Conceptual model

Typically, organizational members’ implementation of ethical advice is contingent upon the ethical climate within the organization [29, 30]. We posit that the organizational caring ethical climate (a climate where organizational members uphold altruistic ideals and consider the interests of other members) plays a moderating role in the relationships between trait mindfulness and both moral self-efficacy and moral courage as well as in the indirect effect of trait mindfulness on ethical voice. Organizational caring ethical climate involves explicit organizational encouragement of caring for others and ethical issue addressing, conveying ethical signals and cues for mindful people that ethical voice is valued and protected. According to the moral decision-making model [23, 24], the context serves as a boundary condition that influences whether an individual engages in moral behavior. Previous studies have proposed that when the environment can provide moral clues, the mindful individual is able to follow the moral clue heuristically, thereby stimulating the individual’s moral decision-making process [29]. Therefore, in the context of an organization that features a highly caring ethical climate, mindful individuals are more likely to generate moral self-efficacy and moral courage via moral cues pertaining to empathy and care, thereby promoting ethical voice. In light of these possibilities, we present a moderated mediation model according to which moral self-efficacy and moral courage mediate the effect of trait mindfulness on ethical voice, whereas the caring ethical climate moderates this effect. Our conceptual framework is illustrated in Fig. 1.

Our article contributes to the literature in several ways. First, while existing research on ethical voice has predominantly adopted a leadership-centered lens, emphasizing how ethical leadership, transformational leadership, or authentic leadership shapes employees’ willingness to express ethical concerns [11, 31], this stream of literature largely overlooks the role of individual-level traits and psychological mechanisms in driving ethical voice. The current research aims to fill this critical gap in the literature by proposing and demonstrating the unique value of trait mindfulness with respect to ethical voice. Thus, our study contributes to the literature by shifting away from the predominant focus of previous research on the contextual factors associated with ethical voice. Second, while most existing research has explored the triggering mechanisms of moral voice based on moral identity theory [11], by using a moral decision perspective to investigate the relationship between mindfulness and ethical voice, the current research provides novel insights and enriches our understanding of the mechanism underlying the relationship between trait mindfulness and ethical voice. Third, by introducing the moderating role of the caring ethical climate in this context, this study generates unique insights by identifying a critical boundary condition of mindfulness and ethical voice.

The remainder of this paper is structured as follows. First, we review the relevant literature and develop our theoretical framework, including hypotheses regarding the relationships between mindfulness, moral self-efficacy, moral courage, caring ethical climate, and ethical voice. Second, we describe the research methodology, including the time-lagged data collection procedure, sample characteristics, measurement tools, and analytical strategies. Third, we present the empirical results of the hypothesis tests. Fourth, we discuss the theoretical and practical implications of our findings, alongside the study’s limitations and directions for future research. Finally, we conclude with a summary of the study’s key contributions and broader insights for organizational ethics management.

### Theoretical framework

#### Moral decision-making theory

This study is grounded in the classic four-stage moral decision-making process, which comprises moral awareness (the recognition of ethical issues), moral judgment (the evaluation of the ethical rightness of actions), moral intention (the commitment to act ethically), and moral action (the actual implementation of ethical initiatives) [24]. We map each core construct to a distinct stage to clarify its unique theoretical role: as a stable dispositional individual difference, mindfulness operates at the moral awareness stage, and its enhanced present-moment attention and unbiased perception enable employees to capture subtle ethical issues and identify moral dilemmas that might otherwise be overlooked [32]; moral self-efficacy focuses on the moral judgment stage, reflecting individuals’ belief in their ability to conduct ethical evaluations and form sound moral judgments, which can strengthen the confidence and accuracy of their moral reasoning [33]; moral courage functions at the moral intention stage, alleviating individuals’ concerns about social or career risks, transforming moral judgments into a firm intention to engage in ethical voice, and bridging the gap between ethical cognition and behavioral commitment; caring ethical climate serves as a moderator in the translation from moral intention to moral action, as a strong caring ethical climate reduces contextual barriers such as peer silence and managerial retaliation, and promotes the conversion of ethical voice intention into actual behavior by reinforcing the legitimacy of moral behavioral intentions [29].

#### Mindfulness and moral self-efficacy

Mindfulness is the consciousness that arises from paying attention to the purpose in the present moment, i.e., without passing judgement on things as they are. It includes two components: (1) attention/awareness of what is experienced in the present (self-regulation of attention) and (2) experiencing processing without judgement, thereby exhibiting sensitivity and openness [34]. Trait mindfulness is believed to provide specific motivation (e.g., self-efficacy) regarding individual behaviour [35, 36]. We postulate that mindfulness may be positively associated with moral self-efficacy. First, mindfulness is believed to facilitate more adaptive self-evaluations by relaxing habitual or automatic cognitive repertoires concerning oneself [37]. Thus, trait mindfulness may help people become more aware of their own self-efficacy by increasing their awareness of the perspective that characterizes any event, including those involving items, people, locations, or ideas. Previous studies have indicated that heavier use of description, acting with awareness, and acceptance without judgement are connected with higher levels of coping self-efficacy [38]. Second, mindfulness reduces the possibility of individuals rationalizing concepts and dismissing possible hazards to their egos, thereby increasing their perceptions of their ability to overcome problems and produce ethical answers [39]. Previous studies have reported that mindfulness may help employees thrive both intellectually and physically, and it can increase their moral self-efficacy by enhancing their physiological conditions [38]. Therefore, we propose the following hypothesis:

- *Hypothesis 1*: Mindfulness is positively related to moral self-efficacy.

#### Mindfulness and moral courage

Moral courage is defined in terms of the fortitude necessary to translate moral intentions into acts the challenges (whether internal or external to the organization) that one encounters during this process [40]. Such courage has been viewed by the positive psychology movement as a key human strength [41]. Mindfulness has many impacts on an individual’s emotions, such as increasing and preserving the individual’s emotional resources [42, 43] and fostering the individual’s moral emotions [44]. We postulate that mindfulness is positively associated with moral courage. First, mindfulness encourages people to be present in the moment and to notice their thoughts and feelings objectively and without judgement [45]. Individuals who exhibit mindfulness may be more likely to identify the emergence of fear and its consequences for their behaviour, thus allowing them to respond by exhibiting moral courage and ethical conduct [46]. Second, according to Baumeister’s self-regulation theory [47], emotion alongside mindfulness, can help steer the individual’s choices by ensuring that long-term concerns are included in the process of moral decision-making. When people become aware of their motives, feelings, thoughts, and desires, they may be able to analyse these factors and determine whether to translate their moral intentions into acts. An understanding of the value of emotional awareness and self-regulation may improve the individuals’ inclination to act courageously and in an ethical manner or his or her possibility of doing so [48]. Notably, a competing theoretical explanation and critical boundary condition exist for the mindfulness–moral courage relationship: in adverse organizational contexts (e.g., punitive climates, retaliation against voice), mindfulness may trigger withdrawal rather than courage. By heightening awareness of situational risks, it could lead employees to choose silence over moral courage when psychological safety is absent. A small number of studies have recently reported that mindfulness is a catalyst for moral courage [49]. Therefore, we propose the following hypothesis:

- *Hypothesis 2*: Mindfulness is positively related to moral courage.

#### Moral self-efficacy and ethical voice

Self-efficacy beliefs are a key predictor of specific motivated actions [33, 50]. Employee voice requires a strong sense of self-efficacy because opposing the status quo entails a high level of psychological risk [51, 52]. MJ Withey and WH Cooper [53] reported that predicted efficacy influences the individuals’ choice to speak up, and perceived efficacy has also been identified as a key feature of whistleblowers. To participate in a behaviour such as voice, it is crucial to be convinced that one “can do” it and is capable of bearing the demands and repercussions associated with such behaviour [51, 52]. A great deal of empirical evidence has suggested certain types of self-efficacy beliefs, such as role breadth self-efficacy, inspire particular behaviours [54, 55]. Moral self-efficacy, which is a type of efficacy belief that is explicitly connected to moral action, is believed to influence ethical voice because the individual’s sense that he or she can effectively handle what is required to achieve moral performance motivates him or her to share his or her genuine concerns regarding moral issues [48]. Moral self-efficacy beliefs that involve the moral intention to engage in desirable team behaviours are likely to result in team extrarole results [10, 48, 56]. Previous studies have reported that moral self-efficacy represents the ease or difficulty involved in performing the moral behaviour of interest and that this factor has a positive effect on ethical voice [26, 31]. Therefore, we propose the following hypothesis:

- *Hypothesis 3*: Moral self-efficacy is positively related to ethical voice.

## Moral courage and ethical voice

Generally, the increased approach and prosocial motives that are linked to moral courage indicate that this characteristic inspires approach-oriented prosocial acts such as those associated with prosocial voice [57]. To take moral action, individuals must know what the “right” judgement entails and possess the fortitude necessary to stand up in the face of adversarial situations, which requires sufficient moral courage to overcome perceived risks while pursuing a moral cause [58, 59]. Moral courage has also been demonstrated to be a crucial link between judgements and ethical conduct in such settings [60]. According to LE Sekerka, RP Bagozzi and R Charnigo [61], facing and resolving ethical challenges as well as confronting obstacles that may prevent one from taking the right action require moral courage; therefore, moral courage is an essential characteristic that is required for ethical behaviour in business settings. Ethical voice, as an extrarole and ethical behaviour, can be cultivated within organizations that encourage employees to raise their concerns regarding ethical issues [62]. According to C Peterson [63], moral courage may compel or allow a person to do what he or she believes is right, regardless of the social or economic consequences.” As a result, it contributes to the consistency of moral intentions and behaviours [64]. In previous studies, scholars have argued that moral courage predicts employees’ likelihood of taking a stand against ethical violations in line with their own moral values [65] as well as their likelihood of taking the right action (e.g., engaging in ethical voice) in their own right [60]. Therefore, we propose the following hypothesis:

- *Hypothesis 4*: Moral courage is positively related to ethical voice.

### The mediating roles of moral self-efficacy and moral courage

We contend that moral self-efficacy and moral courage play mediating roles in the relationship between mindfulness and ethical voice. According to the ethical decision-making model [23, 24], individual and situational factors can jointly influence the ethical decision-making process (such as by promoting moral cognition and moral emotion and thus triggering moral judgement and moral action). Ethical voice refers to the moral behaviour exhibited by an individual who chooses to exercise his or her voice in response to an ethical issue within an organization, and it is the result of an individual’s moral decision-making in response to multiple possible choices [22].Mindfulness helps individuals pay attention to current ethical clues regarding moral self-regulation, thus improving their moral self-efficacy and moral courage; in turn, moral self-efficacy and moral courage drive individuals to engage in ethical voice in organizations. Previous studies have confirmed that moral self-efficacy and moral courage both play key mediating roles in the process of promoting the implementation of ethical voice behaviour [28, 31]. Accordingly, we propose the following hypotheses:

- *Hypothesis 5*: Moral self-efficacy mediates the relationship between mindfulness and ethical voice.
- *Hypothesis 6*: Moral courage mediates the relationship between mindfulness and ethical voice.

#### The moderating role of caring ethical climate

Ethical voice may be risky in situations involving ethical problems and moral flaws. Therefore, an appropriate organizational context may be more likely to cultivate ethical voice among employees [30]. An organization that features a caring ethical climate upholds altruistic ideals and considers the interests of its members [29]. When organizations care for ethical issues, organization members are more likely to pay more attention to the interests of other stakeholders and to consider the impact of their decisions on stakeholders. According to the moral decision-making model [24], contextual and individual factors interact with each other in the context of moral decision-making, and the organizational ethical climate has been proven by previous research to be a key context in which individuals engage in moral behaviour [23]. A caring ethical climate focuses on the well-being of employees, emphasizes the need to treat organizational members with respect and in an ethical manner, and encourages members of the organization to exhibit compassion and help others [66]. Specifically, mindful individuals are able to motivate the moral decision-making process easily by relying on moral cues [29]. Thus, a highly caring ethical climate provides mindful employees with moral clues through empathy and care. In this situation, mindful people are more easily aware such moral clues, which then stimulate their decision-making process alongside their moral self-efficacy and moral courage. In contrast, in a less caring ethical climate (or even in a political climate), mindful individuals lack external cues to guide them; in such a context, individuals lack the confidence and courage necessary to enhance their ethical voice and even exhibit withdrawal and deviation in response to moral matters [67]. Previous research has confirmed that the organizational climate plays a key role in shaping the functions of mindfulness in the workplace [68, 69]. For example, KY Kao, CL Thomas, C Spitzmueller and YH Huang [68] reported that a safety climate can strengthen the effect of trait mindfulness on employees’ safety behaviour.

We previously proposed that trait mindfulness has an indirect effect on ethical voice via moral self-efficacy and moral courage. Our theorizing is limited to the moderating role of a caring ethical climate on the effects of mindfulness on moral self-efficacy and moral courage. We further propose that a caring ethical climate conditionally impacts the indirect effect of mindfulness on ethical voice. Accordingly, we propose the following hypotheses:

- *Hypothesis 7*: Caring ethical climate moderates the positive relationship between mindfulness and moral self-efficacy such that this relationship is stronger at high levels of caring ethical climate than at low levels of a caring ethical climate.
- *Hypothesis 8*: Caring ethical climate moderates the positive relationship between mindfulness and moral courage such that this relationship is stronger at high levels of caring ethical climate than at low levels of caring ethical climate.
- *Hypothesis 9*: Caring ethical climate moderates the positive impact of mindfulness on ethical voice via moral self-efficacy such that this relationship is stronger at high levels of caring ethical climate than at low levels of caring ethical climate.
- *Hypothesis 10*: Caring ethical climate moderates the positive impact of mindfulness on ethical voice via moral courage such that this relationship is stronger at high levels of caring ethical climate than at low levels of caring ethical climate.

This study grounds all hypotheses in a unified moderated mediation model derived from moral decision‑making theory, which links individual dispositions and contextual factors to employee ethical voice. Trait mindfulness acts as the core antecedent, exerting a positive influence on ethical voice both directly and indirectly through moral self‑efficacy and moral courage as parallel mediators. Hypotheses 1–2 posit mindfulness as a predictor of these two mediators, Hypotheses 3–4 connect the mediators to ethical voice, and Hypotheses 5–6 confirm their mediating roles. Caring ethical climate is proposed to strengthen the positive associations between mindfulness and the mediators (Hypotheses 7–8) and to amplify the indirect effects of mindfulness on ethical voice via moral self‑efficacy and moral courage (Hypotheses 9–10). Collectively, these hypotheses form an integrated framework: mindfulness fosters ethical voice by enhancing moral judgment and intention, while a caring ethical climate amplifies these psychological processes, jointly illustrating how individual and contextual factors interact to promote employees’ ethical expression.

## Methods

### Participants and procedure

we collected data by conducting a survey of Chinese work groups from many industries, such as retail, technology, finance, and health care, from December 2023 to May 2024 in Changchun, which is located in northeastern China; participants in this survey were compensated for their participation. We are connected to the human resource (HR) managers of these companies through our personal social networks. We asked the HR managers of the included companies to recruit participants in their organizations. We notified HR managers of the goal and procedure of this study prior to their participation in this research. Before we administered the formal survey, a full list of employees (which was anonymized but included basic demographic information and staff IDs) was collected with the assistance of the HR managers. Respondents were randomly selected from this list. A time-lagged study was conducted at three time points (which were separated by intervals of 1 month each). The questionnaires could be completed either in person or via email. The questionnaires were matched on the basis of researcher-defined codes that were developed before the surveys were administered. Other participants e-mailed their responses to the questionnaire to the researchers. We informed all participants of relevant information regarding the study as well as the purpose and confidentiality of this research. Research has reported that a 1-month time lag significantly reduces common method variance (CMV)-related inflation [70]. The participants rated mindfulness and the caring ethical climates of their organizations at time 1. In the second wave, which took place approximately 1 month later, we obtained participants’ ratings of moral self-efficacy and moral courage, and at time 3, we collected assessments of the ethical voice exhibited by the participants. All participants provided voluntary written informed consent for their participation in this study. To improve the response rate for this research, participants who responded to all three surveys were entered into a random drawing for one of a set of $5 gift cards. In total, 453 participants responded to these questionnaires (response rate: 84.44%). All procedures used in this study were performed in accordance with the ethical standards of the relevant institutional and national research committees and in line with the 1964 Helsinki declaration and its later amendments or comparable ethical standards. This study was approved by the Ethical Committee for Psychological Research of the institution associated with the first author.

In total, 231 respondents in the sample were male, while 222 were female. The average age of the respondents was 28.94 years (SD = 0.80), and respondents’ ages ranged from 24 to 55 years. A total of 272 respondents had obtained a bachelor’s degree, and 8% had obtained a graduate degree. The respondents had an average of 5.63 years of work experience (SD = 1.29) in various industries (e.g., retail, technology, finance, and health care).

### Measures

All items included in the survey were translated from English to Chinese via accepted translation back-translation techniques [71]. To guarantee semantic equivalence between the original and Chinese versions, three bilingual scholars with expertise in organizational ethics and cross-cultural research reviewed the translated items for conceptual consistency, semantic accuracy, and cultural appropriateness. Any inconsistent or ambiguous expressions were revised via panel discussion until full semantic equivalence was achieved, ensuring the Chinese scale retained the original theoretical connotation. The participants rated their levels of agreement with the items included in the survey on a 5-point Likert scale (which ranged from 1 = never to 5 = always).

### Ethical voice

Ethical voice was measured via the 6-item scale developed by JA LePine and L Van Dyne [8]. Sample items include “I would communicate moral issues to others in my group” (Cronbach’s alpha = 0.90).

### Mindfulness

Mindfulness was measured via 6 items developed by KW Brown and RM Ryan [32]. A sample item was “I become so focused on the goal that I want to achieve that I lose touch with what I am doing right now to get there” (reverse coded) (Cronbach’s alpha = 0.89).

### Moral self-efficacy

Moral self-efficacy was measured via 6 items developed by ST Hannah and BJ Avolio [56]. A sample item is “I am confident that I can determine what needs to be done when I face ethical dilemmas” (Cronbach’s alpha = 0.91).

### Moral courage

The 4-item scale for moral courage developed by ST Hannah, BJ Avolio and FO Walumbwa [48] was used to assess organization members’ moral courage. A sample item is “I will confront my peers if they commit an unethical act” (Cronbach’s alpha = 0.89).

### Caring ethical climate

Caring ethical climate was measured via 7 items developed by B Victor and JB Cullen [29]. A sample item is: “I perceive that the concerns of our organization are always what is best for the other person” (Cronbach’s alpha = 0.88).

### Control variables

As indicated in previous studies, such as D Lee, Y Choi, S Youn and JU Chun [31], we controlled for gender, age, educational level, work tenure, and industry type, all of which have theoretical and empirical ties to employee ethical voice. Demographic variables (gender, age, education) were included because they shape employees’ moral cognition, risk aversion, and propensity to engage in ethical voice. Work tenure was controlled as it affects employees’ organizational embeddedness and perception of ethical voice risks. Industry type was controlled to account for industry-level differences in ethical norms and psychological safety for voice.

### Analytical strategy

First, to improve the reliability and accuracy of our hypothesis testing, we employed SPSS software to assess demographic features and correlations among the research variables. Second, confirmatory factor analysis (CFA) was conducted with the assistance of Mplus 7.0 software with the goal of examining the discriminant validity and fit of the measurement model. Following the well-established psychometric standards proposed by C Fornell and DFJJomr Larcker [72], we assessed construct reliability and convergent validity using composite reliability (CR) and average variance extracted (AVE), alongside Cronbach’s alpha. A CR value ≥ 0.70 indicates acceptable construct reliability, while an AVE value ≥ 0.50 suggests satisfactory convergent validity, meaning the construct accounts for more variance in its items than random measurement error. These indicators are systematically reported in the Results section to validate the measurement quality. Third, we used PROCESS models 4 and 7 by AF Hayes [73] and followed the suggestions of KJ Preacher, MJ Zyphur and Z Zhang [74] to validate the hypotheses proposed in this research. To clarify the stepwise variable inclusion logic and enhance transparency, we outline the analytical sequence aligned with our PROCESS models below. For mediation analysis (Model 4), we implemented three steps: (1) testing the direct effect of mindfulness on ethical voice; (2) estimating mindfulness’s effects on moral self-efficacy and moral courage (mediators); (3) adding mediators to the model to assess indirect effects alongside the direct effect. For moderated mediation (Model 7), we then: (4) entered main effects of mindfulness, caring ethical climate (moderator), and mediators; (5) included the mindfulness × climate interaction term to test moderation, followed by simple slope and conditional indirect effect tests. This sequence reflects the regression logic embedded in the PROCESS macro, ensuring clear reporting of how mediators and moderators were integrated sequentially.

We used 5000 bootstrap resamples to determine the indirect effects and obtained 95% confidence intervals. If the confidence intervals did not include 0, then the indirect effect was considered to be significant. We adhered to the recommendations of JR Edwards and LS Lambert [75] and used the simple slope test to examine the moderating effect [76]. We also used the method outlined by KJ Preacher, DD Rucker and AF Hayes [77] to test the conditional indirect impact in further detail.

## Results

### Common method variance

Because the data consulted in this research were acquired via the self-report method, CMV might impact the results of this research [78]. To examine the possibility of CMV, we followed the suggestions of previous research by performing exploratory factor analysis (EFA) via the Harman single-factor test without rotation to investigate the items included in all the scales used in this research. The variance explained by the first factor was 13.84%, i.e., far below the critical threshold of 40%, thus indicating that CMV did not affect the results of this study. We also employed the unmeasured latent method factor (ULMF) approach to investigate CMV [79]. The ULMF statistics suggested a minor enhancement in model fit (*χ*^2^ = 1381.60; *df* = 552; CFI = 0.92; TLI = 0.91; IFI = 0.92; RMSEA = 0.060). The method factor accounted for 16.87% of the overall variance, i.e., less than 0.5 [80–82].

### Confirmatory factor analysis

We performed CFAs with the assistance of Mplus 7.0 to ensure discriminant validity of our study measures (i.e., mindfulness, moral courage, moral self-efficacy, ethical voice, and caring ethical climate). As indicated in Table 1, the results revealed that the five-factor model exhibited a better model fit than did the alternative models (*χ*^2^ = 1391.77, *df* = 552, CFI = 0.92, TLI = 0.91, RMSEA = 0.06). According to the conventional criteria, composite reliability (CR) should be greater than 0.70 to indicate acceptable construct reliability, and average variance extracted (AVE) should exceed 0.50 to demonstrate satisfactory convergent validity. We next investigated the extracted average variance (AVE) and composite reliability (CR) of the variables to assess convergent validity in light of accepted criteria drawn from previous studies [72, 83]. Tables 2 and 3 indicate that the discriminant validity exhibited by each variable included in this study was good.

**Table 1:** Confirmatory factor analysis

Measurement Models	χ2	df	CFI	TLI	IFI	NFI	RMSEA
Five-factor Four-factor (combining mindfulness and caring ethical climate into one factor)	1391.77 1469.43	552 563	0.92 0.90	0.91 0.89	0.92 0.90	0.90 0.89	0.060 0.074
Three-factor (combining mindfulness and caring ethical climate and moral self-efficacy into one factor)	1875.80	565	0.81	0.80	0.81	0.80	0.095
Two-factor (combining mindfulness and caring ethical climate and moral self-efficacy and moral courage into one factor)	3006.12	567	0.61	0.60	0.61	0.60	0.140
One-factor (combining all items into one factor)	4367.92	568	0.52	0.50	0.52	0.50	0.147

*CFI *comparative fit index, *IFI *incremental fit index, *TLI *Tucker-Lewis index, *RMSEA *root mean square error of approximation

**Table 2:** Convergent validity

Constructs	AVE	CR
1.Mindfulness	0.58	0.87
2.Caring ethical climate	0.44	0.84
3. Moral self-efficacy	0.55	0.86
4. Moral courage	0.63	0.87
5.Ethical voice	0.47	0.86

*AVE *average variance extracted, *CR *composite reliability

**Table 3:** Discriminant validity

Constructs	1	2	3	4	5
1.Mindfulness	0.76				
2.Caring ethical climate	0.11*	0.66			
3. Moral self-efficacy	0.49**	0.12^**^	0.74		
4. Moral courage	0.54**	0.14^**^	0.38^**^	0.79	
5.Ethical voice	0.50**	0.16^**^	0.42^**^	0.49^**^	0.68

## Results

### Descriptive statistics and correlations

Table 4 presents the means, standard deviations, sample sizes, and intercorrelations for all the variables included in this study. The results of our regression analyses are presented in Table 4. The results indicated that mindfulness (time 1) was significantly and positively associated with moral self-efficacy (time 2) (*r* = 0.49, *p* < 0.01), moral courage (time 2) (*r* = 0.54, *p* < 0.01), and ethical voice (time 3) (*r* = 0.50, *p* < 0.01). In addition, moral self-efficacy (time 2) was positively related to ethical voice (time 3) (*r* = 0.42, *p* < 0.01). Moral courage (time 2) was positively related to ethical voice (time 3) (*r* = 0.49, *p* < 0.01). Caring ethical climate was positively correlated with moral self-efficacy (time 2) (*r* = 0.12, *p* < 0.001), moral courage (time 2) (*r* = 0.14, *p* < 0.01), and ethical voice (time 3) (*r* = 0.16, *p* < 0.01). These correlation findings provided preliminary validation of our hypotheses.

**Table 4:** Results of descriptive statistical analysis

Variables	M	SD	1	2	3	4	5	6	7	8	9
1. Gender	0.51	0.50	1								
2. Education	3.03	1.21	0.00	1							
3. Age	2.61	0.80	-0.08	-0.05	1						
4. Tenure	2.68	1.29	0.08	-0.01	-0.03	1					
5.Mindfulness (Time 1)	3.54	0.66	0.04	-0.03	0.02	0.04	(0.89)				
6.Caring ethical climate (Time 1)	3.42	1.67	0.00	-0.01	0.05	-0.00	0.11^*^	(0.88)			
7. Moral self-efficacy (Time 2)	3.75	0.67	0.02	-0.00	0.09^*^	0.04	0.49^**^	0.12^**^	(0.91)		
8. Moral courage (Time 2)	3.63	0.73	0.00	-0.11^*^	0.00	0.06	0.54^**^	0.14^**^	0.38^**^	(0.89)	
9.Ethical voice (Time 3)	3.44	0.87	0.00	-0.01	0.05	0.05	0.50^**^	0.16^**^	0.42^**^	0.49^**^	(0.90)

*N* = 453; Cronbach’s alpha reliabilities displayed on the diagonal; * *p* < 0.05. ** *p* < 0.01.*** *p* < 0.001. (Two-tailed test)

### Hypothesis testing

We first tested the direct effects and indirect effects between mindfulness and ethical voice. As presented in Table 5, mindfulness positively predicted moral self-efficacy (*β* = 0.49, *p* < 0.001), moral courage (*β* = 0.54, *p* < 0.001), and ethical voice (*β* = 0.50, *p* < 0.001). Moral self-efficacy positively predicted ethical voice (*β* = 0.22, *p* < 0.001), and moral courage positively predicted ethical voice (*β* = 0.31, *p* < 0.001). Therefore, Hypotheses 1–4 were supported.

**Table 5:** Moderated mediation analysis

Variables	Moral self-efficacy		Moral courage				Ethical voice		
	Model 1	Model 2		Model 3	Model 4	Model 5		Model 6	Model 7
Intercept	1.77^***^	2.34^***^		1.64***	2.27^***^	0.97^***^		0.45*	0.36
Age	-0.00	-0.00		-0.07*	-0.07^*^	-0.05		-0.05	-0.03
Gender	0.01	0.01		-0.09	-0.08	0.00		0.00	0.03
Education	0.08*	0.08		-0.01	-0.02	0.04		0.02	0.05
Tenure	0.02	0.02		0.07	0.08	0.06		0.05	0.03
Mindfulness	0.49^***^	0.31^***^		0.54^***^	0.35^***^	0.50^***^		0.39^***^	0.33^***^
Moral self-efficacy								0.22^***^	
Moral courage									0.31^***^
Caring ethical climate		-0.42^*^			-0.42^*^				
Mindfulness× Caring ethical climate		0.53^*^			0.54^**^				
*R* ^2^	0.25	0.26		0.30	0.32	0.26		0.30	0.33
Adjusted *R*^2^	0.24	0.25		0.30	0.31	0.25		0.29	0.32
*F*	28.73^***^	21.77^***^		37.90***	28.73^***^	30.08^***^		30.37^***^	34.94^***^

*N* = 453; * *p* < 0.05. ** *p* < 0.01.*** *p* < 0.001. (Two-tailed test)

Second, the results of the mediation tests are presented in Table 5. A bootstrapping-based process of analysis (*n* = 5,000) [PROCESS, model 4; 73] was used to examine the indirect effect. Mindfulness was associated with increases in ethical voice via increases in moral self-efficacy (estimate = 0.17, SE = 0.06, 95% CI= [0.071,0.322]) and moral courage (estimate = 0.25, SE = 0.06, 95% CI= [0.0831, 0.392]); therefore, Hypotheses 5 and 6 were fully supported.

Third, we examined a moderated mediation model as part of our study. We followed the guidelines suggested by JR Edwards and LS Lambert [75] by using the PROCESS model (Model 7). As indicated in Table 5, the results indicated that the interactions between mindfulness and caring ethical climate were significant with respect to the relationships between mindfulness and moral self-efficacy (*β* = 0.53, *p* < 0.05) (Model 2 in Table 5) and between mindfulness and moral courage (*β* = 0.54, *p* < 0.01)(Model 4 in Table 5). We further tested this moderating effect by performing a simple slope test and generated a corresponding figure [84], as illustrated in Fig. 3. The results indicated that mindfulness was strongly significantly and positively related to moral self-efficacy in a strong caring ethical climate (*β* = 0.74, SE = 0.05, *p* < 0.001), comparing with a weak caring ethical climate (*β* = 0.41, SE = 0.06, *p* < 0.001); therefore, Hypothesis 7 was supported. Also, as illustrated in Fig. 3, the results indicated that mindfulness was strongly significantly and positively related to moral courage in a strong caring ethical climate (*β* = 0.63, SE = 0.05, *p* < 0.001), compared with a weak caring ethical climate (*β* = 0.33, SE = 0.05, *p* < 0.001); therefore, Hypothesis 8 was supported.

**Fig. 2:**
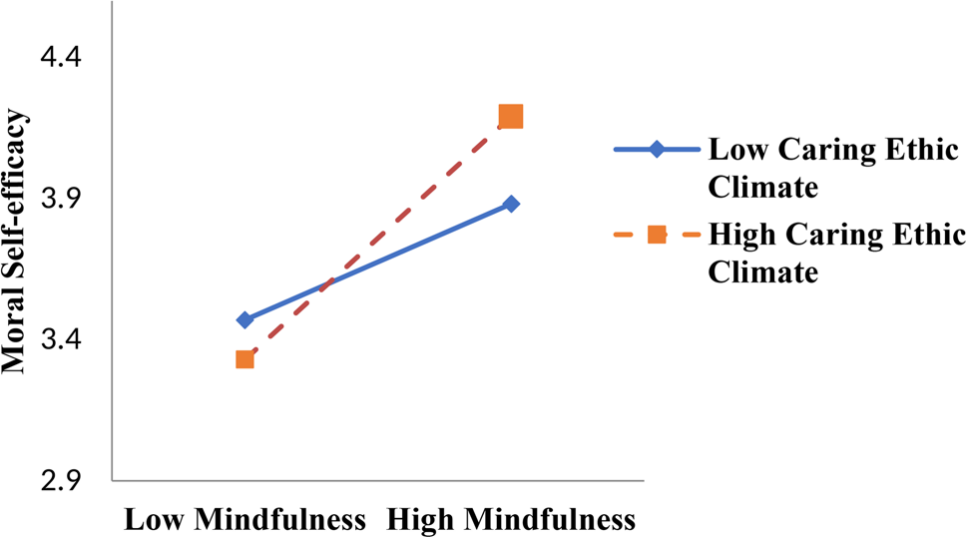
Moderating effect of caring ethical climate on the relationship between mindfulness and moral self-efficacy

**Fig. 3:**
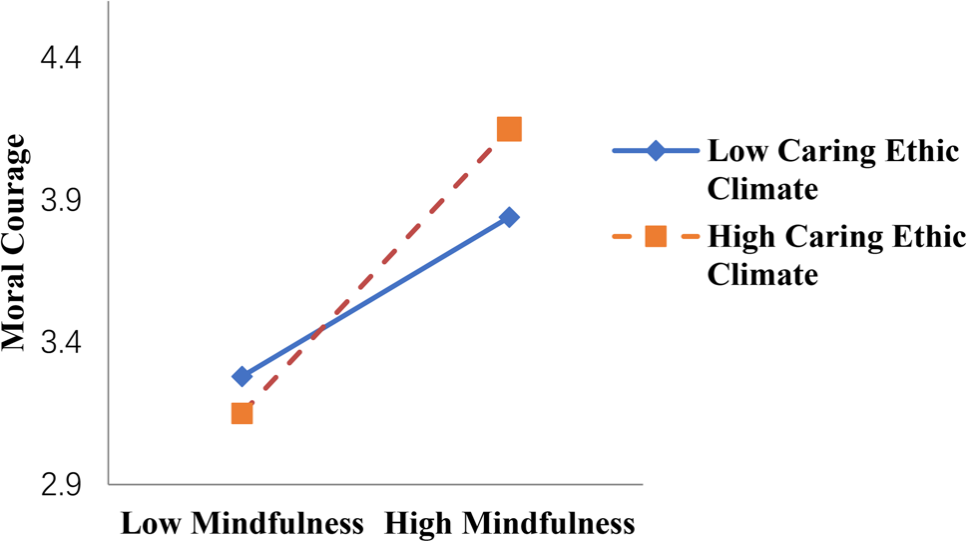
Moderating effect of caring ethical climate on the relationship between mindfulness and moral courage

**Fig. 4:**
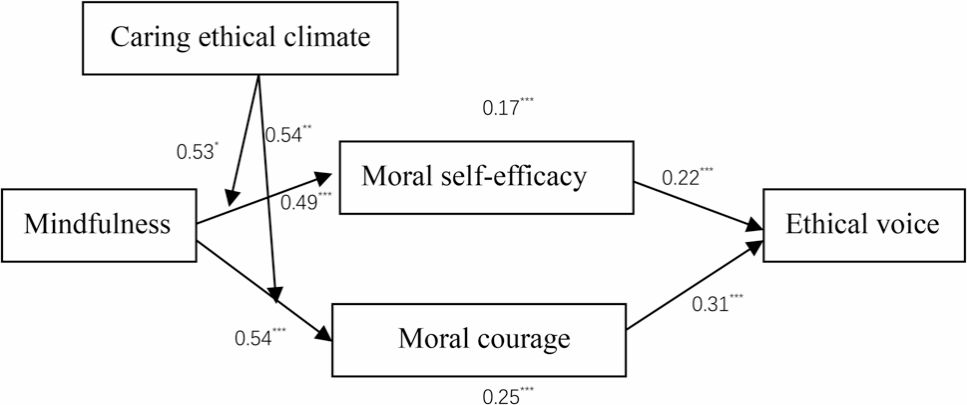
Structural Model

Furthermore, we tested the conditional indirect effect posited in the model. As illustrated in Table 6, the empirical results revealed that the indirect effect of mindfulness on ethical voice via moral self-efficacy was significant and positive in a strong caring ethical climate (indirect effect = 0.19, 95% CI= [0.060, 0.371]), comparing with a weak caring ethical climate (indirect effect = 0.10, 95% CI=[0.030, 0.200]). The estimated and bootstrapped confidence intervals of the index of moderated mediation (*r* = 0.02, 95% CI = [0.001, 0.068]) thus supported Hypothesis 9. As illustrated in Table 7, these empirical analyses also revealed that the indirect effect of mindfulness on ethical voice via moral courage was significant and positive in a strong caring ethical climate (indirect effect = 0.27, 95% CI = [0.130, 0.430]), unlike in a weak caring ethical climate (indirect effect = 0.15, 95% CI = [0.070, 0.270]). The estimated and bootstrapped confidence intervals of the index of moderated mediation (*r* = 0.03, 95% CI = [0.001, 0.081]) thus provided support for Hypothesis 10. The full structural model was shown in Fig. 4.

**Table 6:** Conditional indirect effects of mindfulness on ethical voice via moral self-efficacy

Moderator	Level	Effect	Boot SE	Boot *p*	CI
Caring ethical climate	Low (-1 *SD*)	0.10	0.04	0.00	[0.030, 0.200]
	High (+ 1 *SD*)	0.19	0.07	0.00	[0.060, 0.371]

*N* = 453, *CI *confidence interval. Bootstrapping repetitions. *N* = 5000

**Table 7:** Conditional indirect effects of mindfulness on ethical voice via moral courage

Moderator	Level	Effect	Boot SE	Boot *p*	CI
Caring ethical climate	Low (-1 *SD*)	0.15	0.05	0.00	[0.070, 0.270]
	High (+ 1 *SD*)	0.27	0.07	0.00	[0.130, 0.430]

*N* = 453, *CI *confidence interval. Bootstrapping repetitions. *N* = 5000

### Robustness test

Considering potential alternative explanations in the competitive model, we first tested the proposed competitive model for chained mediating relationships such as mindfulness– moral self-efficacy→ moral courage→ethical voice and mindfulness– moral self-efficacy→ moral courage→ethical voice. We further employed Hayes’ (2017) PROCESS macro (model 6) to test chained mediating relationships. The results indicated that both the chain mediating relationships of Mindfulness-moral self-efficacy-moral courage-ethical voice (estimate = 0.02, SE = 0.015, 95% CI= [0.020, 0.050]) and Mindfulness-moral courage-moral self-efficacy-ethical voice (estimate = 0.01, SE = 0.01, 95% CI= [0.001, 0.042]) were significant. Notably, both chained mediation effect sizes were substantially small and negligible in practical and theoretical terms: their point estimates are far lower than the core parallel mediation effects of moral self-efficacy (estimate = 0.17) and moral courage (estimate = 0.25) from our primary mode. These chained paths represent only weak secondary mechanisms, and their statistical significance does not alter the theoretical logic, empirical conclusions, or structural specification of our proposed moderated parallel mediation model, which remains the dominant and theoretically grounded explanatory framework.

## Discussion

This study explores how and why mindfulness affects ethical voice building on the basis of ethical decision model. Consistent with our predictions, our findings indicate that mindfulness has positive effects on moral self-efficacy, moral courage, and ethical voice. In addition, mindfulness positively impacts ethical voice by increasing moral self-efficacy and moral courage. We also identify a caring ethical climate as a boundary condition that strengthens the relationship between mindfulness and moral self-efficacy as well as the relationship between mindfulness and moral courage; it also strengthens the indirect effect of mindfulness on ethical voice.

### Theoretical contributions

Our research has several important theoretical implications. First, the empirical findings of our study reveal that mindfulness significantly and positively impacts ethical voice, thereby answering the call for researchers to extend the nomological network of ethical voice [30]. We can explain this result by reference to the fact that mindful employees are more likely to acknowledge moral dilemmas and to be more conscious of ethical considerations when making the decision to engage in ethical voice behaviours. The findings broadly support the conclusions of previous studies on the positive effects of mindfulness on ethical decision-making and ethical behaviour [16, 19]. While most recent studies have focused on the contextual antecedents of ethical voice (e.g., leadership or organizational climate), the ways in which individual factors affect ethical voice have been overlooked to some degree [31, 85]. Thus, our results contribute new knowledge to the ethical voice literature and extend previous studies by revealing the positive link between mindfulness and ethical voice. The strong effects of mindfulness in the Chinese organizational context are highly associated with local cultural traits. High power distance and the cultural norm of “restrained communication” in Chinese workplaces often lead employees to overlook ethical cues and suppress moral expression, and mindfulness’ core capacity of present-moment awareness precisely offsets this deficiency, which explains its robust predictive effects (*β* = 0.49 ~ 0.54). In addition, although previous research has focused on the positive relationship between mindfulness and voice behaviour [21], our study reconfirms the role of mindfulness as a moral compass in the workplace, which is based on the relationship between mindfulness and ethical voice.

Second, by identifying moral self-efficacy and moral courage as mediators of the relationship between mindfulness and ethical voice, our findings provide new insights into the mechanisms by which mindfulness affects ethical voice. According to the moral decision model [23, 24], we can explain these results by reference to the fact that mindfulness helps individuals develop a sense of moral self-efficacy and moral courage that enables them to navigate moral dilemmas successfully; in turn, this process facilitates their moral action (e.g., ethical voice). These results are in line with previously reported empirical evidence that has highlighted the mediating roles played by moral self-efficacy and moral courage in this context [28, 31]. Nevertheless, these findings contribute to the ethical voice literature, which has focused predominantly on the mechanism underlying the relationship between context variables and ethical voice [11, 31]. While most studies on this topic have used a social cognitive theoretical framework to open the black box of ethical voice [11], our findings integrate two such mechanisms (i.e., cognitive and emotional) by using an ethical decision-making theoretical framework to explain the effect of mindfulness on ethical voice. Thus, these findings contribute to moral decision theory and provide a new perspective on ethical voice.

Finally, our research contributes to the ethical voice literature by identifying caring ethical climate as a boundary condition with regard to the effects of mindfulness on moral courage and moral self-efficacy. In line with the moral decision model [23, 24], these findings confirm that caring ethical climate and mindfulness interact with each other to impact the moral decision-making process. Our findings extend the conclusions of previous research on this topic and contribute to the existing body of knowledge regarding ethical voice by confirming that a caring ethical climate may also serve as a vital moderator of the mindfulness-ethical voice relationship. Furthermore, although few previous studies have reported that the moral climate can moderate the relationship between mindfulness and ethical behaviour [29], the conclusions of this study are consistent with and to some extent go beyond those of previous studies. Collectivist cultural values align with the essence of a caring ethical climate, further amplifying its moderating effects. While the cultural norm of “valuing hierarchical interpersonal harmony” in Chinese workplaces would otherwise constrain the behavioral translation of moral courage, a caring ethical climate effectively mitigates this cultural constraint, enabling the moral psychological mechanisms triggered by mindfulness to translate into actual ethical voice behaviors. These moderating effects can provide us with a deeper understanding of why mindfulness affects ethical voice, thereby offering us an understanding of the boundary conditions under which mindfulness operates in the context of ethical behaviour.

### Practical implications

Our findings have several practical implications for managers. First, in light of the positive effects of mindfulness on ethical voice—with mindfulness strongly predicting moral self-efficacy (*β* = 0.49, *p* < 0.001) and moral courage (*β* = 0.54, *p* < 0.001), and directly influencing ethical voice (*β* = 0.50, *p* < 0.001)—organizations should prioritize mindfulness cultivation in personnel practices. Although trait mindfulness is a dispositional difference, its high effect sizes on core moral psychological mechanisms justify investing in structured mindfulness training. Such programs reinforce the core attentional and non-judgmental components of trait mindfulness (rather than only eliciting transient mindful states). For example, organizations can design standardized interventions (e.g., weekly meditation sessions, mindful leadership workshops, daily present-moment awareness micro-practices) for all employees, as the robust predictive power of mindfulness indicates these efforts will effectively lay the foundation for ethical voice.

Second, to leverage the positive effect of moral self-efficacy on ethical voice (β = 0.22, *p* < 0.001) and its conditional indirect role—with the indirect effect of mindfulness via moral self-efficacy increasing from 0.10 (weak caring ethical climate) to 0.19 (strong caring ethical climate)—organizations should integrate targeted moral self-efficacy development into ethics initiatives. HRM departments can use scientific questionnaires or structured conversations to assess employees’ moral self-efficacy, then design scenario-based training that simulates real-world ethical dilemmas, provides guided feedback on decision-making, and highlights internal ethical voice successes. This tailored approach aligns with the observed effect size, ensuring interventions directly address the moderate but meaningful role of moral self-efficacy in driving ethical voice.

Third, given that moral courage exerts a stronger direct effect on ethical voice (β = 0.31, *p* < 0.001) than moral self-efficacy, and its indirect effect amplifies more substantially under strong caring ethical climates (0.27 vs. 0.15 in weak climates), organizations should prioritize cultivating moral courage through contextual supports. Establishing peer support groups for ethical advocacy, implementing explicit non-retaliation policies to protect voice behavior, and providing leadership training that models courageous ethical actions will directly target the mechanism with the larger observed effect. These measures mitigate the risks of ethical voice, allowing the strong predictive power of moral courage to translate into tangible behavioral outcomes.

Fourth, considering the caring ethical climate’s robust moderating role—strengthening mindfulness-moral self-efficacy (*β* = 0.53, *p* < 0.05) and mindfulness-moral courage (*β* = 0.54, *p* < 0.01) links, and boosting indirect effects via both mediators—managers must proactively foster this climate. Specifically, in strong caring climates, mindfulness’s association with moral self-efficacy (*β* = 0.74 vs. 0.41 in weak climates) and moral courage (*β* = 0.63 vs. 0.33 in weak climates) is markedly enhanced. Organizations can adopt practices like workplace mental health training, stress management programs (preventive care), and emotional support/crisis intervention teams (assistive care) to build this climate, as the observed effect sizes confirm it acts as a critical “amplifier” of core ethical voice mechanisms.

### Limitations

Despite the many theoretical and managerial implications of this study, several limitations of this research also highlight notable opportunities for future studies. First, although we adopted a time-lagged research design to alleviate the issue of common method variance to a certain extent, all variables were measured via self-reports from the same respondents. Specifically, ethical voice, as a behavior with obvious prosocial and moral attributes, is prone to social desirability bias—respondents may overreport their ethical voice behavior to conform to social expectations or organizational ethical norms, which may inflate the observed relationships between variables. Future research can enhance the validity and robustness of findings by collecting multi-source data, such as adopting supervisor-rated or peer-rated scales to measure ethical voice, which can effectively reduce the interference of social desirability bias and single-source bias. Second, all the constructs included in our study were measured through self-reports. Although our study used temporal separation when measuring these variables, this approach did not necessarily rule out the possible impacts of CMV. Future studies should consider the use of paired data and enhance the causal rigor of research on this topic by employing a variety of methods, such as longitudinal studies or experimental methodologies. Third, we focused only on the mediating role of moral self-efficacy and moral courage on the basis of the moral decision-making framework; thus, it remains possible that other mediating mechanisms may explain this relationship. For example, mindfulness is a key resource in this context; according to COR (Conservation of Resource) theory, this key individual resource may contribute to the emergence of a resource spiral that can motivate employees to engage in ethical voice behaviours. Future research could consider analyzing the mindfulness-ethical voice relationship based on COR theory. At the same time, a number of control variables may also exert an impact on our model. We therefore call for the inclusion of more control variables (e.g., the Big Five personality traits, ethical leadership) in future studies to enhance the robustness of the model. We also call for future research to focus on exploring potential downsides of ethical voice for mindful employees (e.g., burnout, retaliation), as well as nonlinear effects of mindfulness (e.g., excessive nonjudgment leading to moral disengagement). Finally, our study is an empirical study that focuses on Chinese cultural scenarios by reference to a sample of employees recruited from Chinese companies. Chinese and Western cultural contexts exhibit significant differences (e.g., collectivism vs. individualism), and the inclusion of cultural contextual variables (e.g., power distance or traditionalism) could be considered in future research with the goal of exploring the boundary conditions associated with the mindfulness-ethical voice relationship in greater depth.

## Conclusion

This study empirically tests a moderated mediation model to explore how and when mindfulness drives employee ethical voice. Findings confirm mindfulness positively predicts ethical voice via parallel mediation of moral self-efficacy and moral courage, with caring ethical climate strengthening these pathways by boosting mindfulness-mediator links. The results advance ethical decision-making theory by unpacking micro-level psychological processes (from moral awareness to judgment/intention) and integrating individual traits with situational factors, extending the classic four-stage model. For organizational ethics research, it complements leadership-centered frameworks, highlighting individual moral resources and their context interaction to expand ethical voice antecedent studies. Practically, cultivating mindfulness, moral traits, and a caring ethical climate enhances organizational ethical governance. This work paves the way for interdisciplinary research at the intersection of organizational behavior, moral psychology, and mindfulness studies—future inquiries could explore neurocognitive mechanisms, integrate clinical mindfulness interventions with ethics training, or examine cross-cultural contingencies. Despite limitations (Chinese sample, individual-level data), the findings offer a new lens for workplace ethical behavior and guide integrated research on individual traits, psychology, and context.

## Data Availability

Data available on request from the corresponding author.
